# Can Machine Learning classifiers be used to regulate nutrients using small training datasets for aquaponic irrigation?: A comparative analysis

**DOI:** 10.1371/journal.pone.0269401

**Published:** 2022-08-16

**Authors:** Sambandh Bhusan Dhal, Muthukumar Bagavathiannan, Ulisses Braga-Neto, Stavros Kalafatis

**Affiliations:** 1 Department of Electrical and Computer Engineering, Texas A&M University, College Station, Texas, United States of America; 2 Department of Soil and Crop Sciences, Texas A&M University, College Station, Texas, United States of America; Universiti Malaysia Pahang, MALAYSIA

## Abstract

With the recent advances in the field of alternate agriculture, there has been an ever-growing demand for aquaponics as a potential substitute for traditional agricultural techniques for improving sustainable food production. However, the lack of data-driven methods and approaches for aquaponic cultivation remains a challenge. The objective of this research is to investigate statistical methods to make inferences using small datasets for nutrient control in aquaponics to optimize yield. In this work, we employed the Density-Based Synthetic Minority Over-sampling TEchnique (DB-SMOTE) to address dataset imbalance, and ExtraTreesClassifer and Recursive Feature Elimination (RFE) to choose the relevant features. Synthetic data generation techniques such as the Monte-Carlo (MC) sampling techniques were used to generate enough data points and different feature engineering techniques were used on the predictors before evaluating the performance of kernel-based classifiers with the goal of controlling nutrients in the aquaponic solution for optimal growth.[27–35]

## Introduction

The food production challenges that the world faces on a daily basis due to globalization and rapid industrialization, have led to increased applications of Aquaponics [1, 2] as a viable alternative to traditional agricultural techniques for improving sustainable food production, given its efficient and sustainable method of water management. The environmental, economic, social, and ethical aspects of these techniques have been of great focus lately, and the varieties of food products emerging from these techniques have been of keen interest. Another major advantage of aquaponics over conventional farming techniques is that it utilizes only 2 to 10% of the water required in traditional vegetables or crop production and has the potential to produce 10 times more output without the use of harmful chemicals and pesticides [3]. There have been a few studies in which laboratory set-ups have been used to optimize nutrients for growing plants in hydroponic environments [4] through controlled set-ups but not a lot has been done to implement them on a larger scale. Although aquaponics has been a topic of research for nearly two decades, very little has been done to automate the process of nutrient control for optimal growth of both fish (the key source of nutrients for an aquaponic farm) and plants in a commercial set-up. All the work until now has been focused on implementing various data-analysis techniques to optimize yield in small controlled set-ups which have been highlighted in the next paragraph.

In the last few years, there have been some advancements in the field of Smart Aquaponics where the environmental, as well as the plant growth parameters, have been monitored using vision-based approaches in a controlled IoT environment. In [5], Arvind et. al. proposed an approach to automatically control the dynamics of the aquaponic system by using an autoML algorithm to improve plant and fish growth and help monitor the system using a cloud platform. The sensor data was collected ten times every day and the fish count was extracted using the R-CNN instance segmentation which was used as a feature to train the algorithm. Similarly, in [6], an IoT-based real time sensing and actuation system has been designed to control the nutrients in an aquaponic set-up depending on the output of a pre-trained ML algorithm which outputs the appropriate nutrient concentrations according to the season in which the lettuce was grown. In [7], features were extracted from lettuce leaves in a smart aquaponic set-up and a comparative study of the three ML estimators: K-Nearest Neighbor (KNN) [8], Logistic Regression [9], and Linear Support Vector Machine (L-SVM) [10] was conducted to detect the diseases that the crop can incur in its lifetime. However, very little research has been done to automate the control of nutrients in aquaponic solutions. The objective of this work is to suggest a recommendation system for regulation of certain chemical nutrients in the aquaponic solution using Machine Learning (ML).Sodium, bicarbonate and chloride concentrations in the aquaponic solution are used as inputs along with the month in which these observations were recorded, and a set of rules have been suggested for optimal growth of both plants and fish in a single set-up.

One of the major limitations that is faced while designing an intelligent system for regulating the nutrient parameters in aquaponic solutions is the lack of data. This study was addressed in [11] where Dhal et al. used Bolstered Error estimation techniques in conjunction with many linear and non-linear classifiers to find the ideal classification technique for regulating nutrients in coupled aquaponic set-ups using small datasets as training datasets. To overcome this, proper feature selection techniques are required. Joundi et al. [12] used an integrated ML approach applying Recursive Feature Selection with Cross-validation (RFECV) which incorporated Linear SVC, Random Forest Classifier and ExtraTressClassifier to select robust features as per their feature importance for ischemic stroke detection. In [13], Chen et al. proposed XGBoost to reduce feature noise and perform dimensionality reduction through gradient boosting and used average gain as an estimate to improve protein-protein interactions. Another important consideration while designing an ML approach with dearth of data is data augmentation by generating synthetic data points. This was addressed by Dahmen et al. in [14] using SynSys, an ML-based synthetic data generation method to generate synthetic time-series data that is composed of nested sequences using hidden Markov models and regression models that are trained on real datasets. Similarly, in [15], Radford M. Neal proposed many techniques of probabilistic inference using Markov-Chain Monte-Carlo methods where the underlying structure of the existing data was used to compute the mean and covariance matrices to generate synthetic data.

Another issue when making inferences with small datasets is the problem of imbalanced classes which one may encounter while making inferences both in the case of supervised and unsupervised learning. In [16], Beckmann et al. demonstrated the efficiency of KNN under-sampling as a technique for creating a balance between the majority and minority classes. Similarly, in [17], the problems of missing values, class imbalance, and high dimensionality in the case of small datasets as well as how under-sampling the majority class provides better sensitivity have been addressed.

Lastly, before deciding on the classifier that would achieve the best classification accuracy, visualizing the data can be useful. In [18], Nasser et al. demonstrated Kernel PCA as a visualization tool by looking at the scatter plot of the projected data and distinguishing different clusters within the original data. Similarly, in [19], Abid et al. proposed contrastive PCA as a tool to identify low-dimensional structures in datasets where data has been collected under different conditions. Next, before doing a comparative study of the classifiers at hand, a study of the different feature engineering techniques can be considered as it may prove useful in improving the classification accuracy of the dataset. Elaborating further on this, Tsagris et al. in [20] proposed a method of data-based power transformation for compositional data by neglecting the compositional constraint and applying standard multivariate data analysis, or by applying logs of the ratios of the components to transform the data. Similarly, in [21], Bogner et al. conducted a Normal Quantile Transformation (NQT) [22, 23] in many hydrological and meteorological applications to make the observed and simulated data conform to Gaussian distribution patterns. In the end, a comparative study of several deterministic kernel-based linear and non-linear classifiers like the Adaboost classifier [24], Gradient Boosting classifier [25], and Linear Support Vector Machine (L-SVM) [26] can be considered to determine the ideal classifier for inferencing on these small datasets for optimizing aquaponic water management.

Following the steps mentioned above in the pipeline, it would be possible to achieve the main aim of the study which is to determine the optimal approach that should be used for nutrient optimization in aquaponic systems. This study can also be used to draw inferences in domains where the size of the dataset is very small.

## Methodology

The dataset used in this case for analysis was recorded from three different aquaponic facilities in East-Central Texas, one from each county: Grimes, Brazos, and Caldwell. The data was collected over the course of a year from June 2020 to June 2021, roughly every week, and was sent to the Soil, Water, and Forage Testing Laboratory Facility at Texas A&M University, College Station, TX for nutrient profiling. Two samples were collected from each of these aquaponic facilities, one from the fish tank and the other from the chamber where the plants were grown. The data collected from both of these chambers were appended onto a single dataset and various data analysis techniques like selecting the optimal features, generating synthetic data, engineering the existing features, and choosing the optimal classifier were used to make a single ML model that can be used for the entire aquaponic system to automate the growth of plants and ensure optimal yield.

In Table 1, how each nutrient was extracted from the aquaponic solution is described in detail.

**Table 1:** Method for measurement of chemical parameters used in Texas A&M Soil, Water and Forage Testing Laboratory, College Station, TX [27–35].

Sl. No.	Name of the Chemical Components	Method of Measurement
1	Calcium, Magnesium, Sodium, Potassium, Boron and heavy metal concentrations (Iron, Zinc, Copper, and Manganese) [All of these measured in ppm]	Inductively Coupled Plasma Analysis (ICP Analysis)
2	Carbonate and Bicarbonate concentrations [ppm]	Acid titration using sulfuric acid
3	Chloride concentration [ppm]	Ion chromatography method
4	Nitrate concentration [ppm]	Reduction to nitrates using a cadmium column followed by spectrophotometric measurement
5	pH	Using hydrogen ion-selective electrode
6	Conductivity (measured in umhos/cm)	Using a conductivity probe

**Fig 1:**
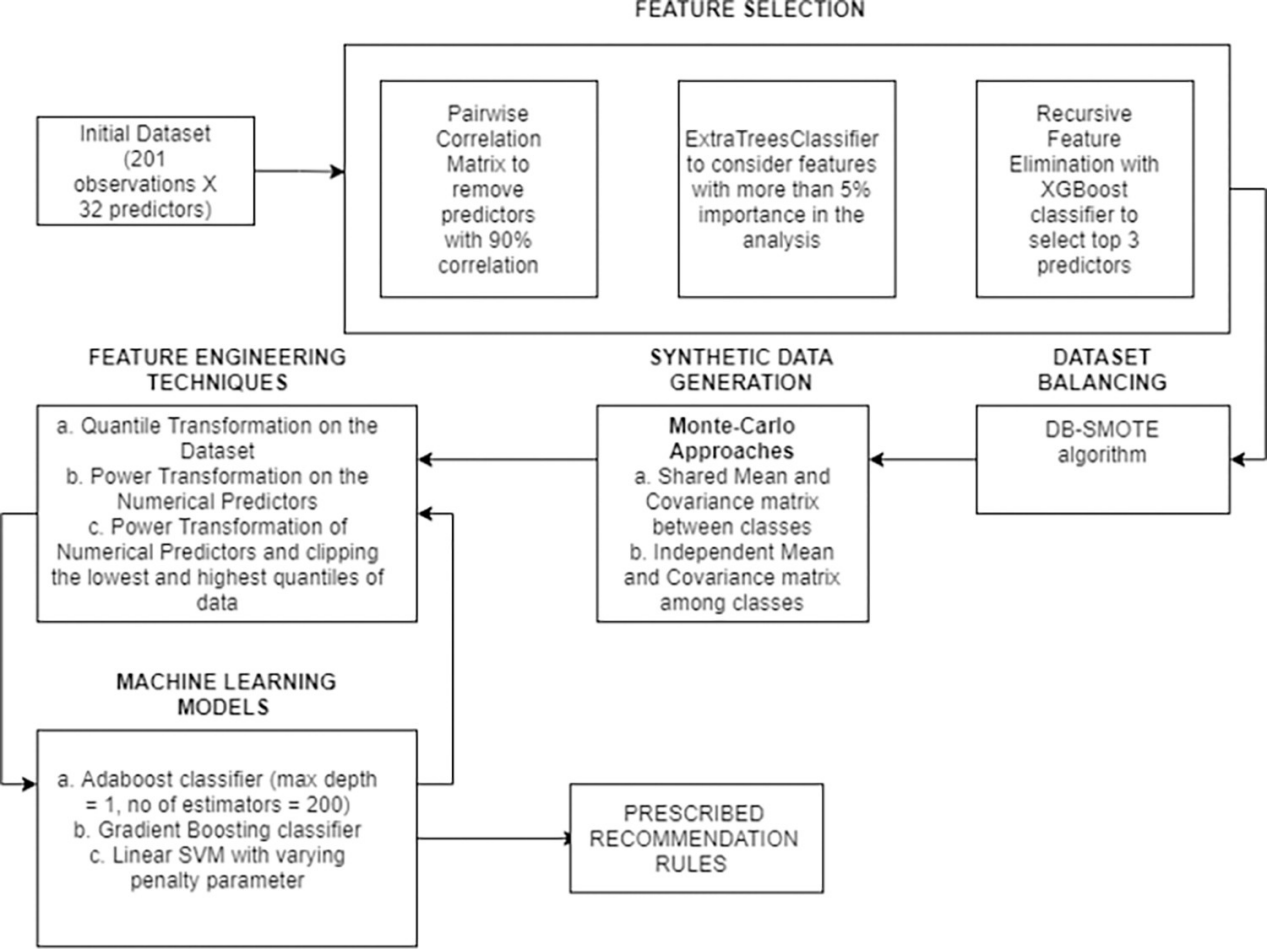
A pipeline of the approach used in the paper for prescribing recommendation rules.

These parameters were coupled with some intrinsic chemical properties of the aquaponic solution and weather parameters for each date when these observations were recorded, to develop a complete dataset which is described in the next section. A comprehensive overview of the approach used in the paper has been stated in Fig 1 below.

### Construction of the dataset

The initial dataset used in this case had a total of 201 observations and 32 predictors. Eleven chemical concentrations have been used as predictors in this case: calcium, magnesium, sodium, potassium, boron, carbonates, bicarbonates, sulfate, chlorides, nitrates, and phosphorus (all of them measured in ppm.); 8 chemical properties of the aquaponic solution: pH, conductivity (umhos/cm), two measures of hardness (one measured in grains CaCO3/gallon and other measured in ppm CaCO3), alkalinity (ppm CaCO3), Total Dissolved Salts (ppm), SAR and Charge Balance; and 4 heavy metal concentrations: Iron, Zinc, Copper and Magnesium (all measured in ppm.).A total of 5 weather predictors for each greenhouse were also appended to the dataset: Wind speed (miles per hour), Temperature (K), Humidity (%), Pressure (mm) and Precipitation (inch).

In addition to this, a total of 4 categorical predictors were also used. The month in which the data were recorded was grouped into 5 categories for analysis. The observations recorded from January through March, April through May, June through August, September through October, and November through December were categorized into Category 1,2,3,4 and 0 respectively and were stored as MONTH_CLASS in the dataset. Similarly, the county in which the data was recorded was one-hot encoded into three categories where Brazos, Caldwell, and Grimes counties were stored as PLACE_CLASS_0, PLACE_CLASS_1, and PLACE_CLASS_2 respectively.

### Feature selection

As stated above, making inferences on a small dataset with a large number of predictors does not produce accurate classifiers, due to the Curse of Dimensionality [36]. For this reason, various feature selection techniques were used to reduce the size of the dataset to 7 predictors. After using the pairwise correlation matrix to remove the chemical predictors which had significant correlation among them, the ExtraTreesClassifier with 250 estimators was used to find the feature importance of these attributes and eliminate the predictors which had less than 5% importance in the analysis. Finally, the XGBoost algorithm with Recursive Feature Elimination was used to rank the predictors and select the top chemical predictors which were coupled with categorical predictors for the entire analysis.

### Dataset balancing and synthetic data generation

As the entire dataset was initially treated as an unsupervised approach using K-Means to cluster them, there is a high probability of an imbalance in the classification of the data due to the extremely small size of the dataset. That is why, the DB-SMOTE algorithm was used to create more samples from the minority class to have a balance in the dataset before generating synthetic data.

For the generation of synthetic data, two variants of the Monte-Carlo (MC) sampling technique were used. As the entire approach is considered as a binary classification problem, the first case of synthetic data generation uses a method in which a separate mean and covariance matrix is generated for each class, sub-categorized by the MONTH_CLASS in which the observations were recorded. The second case of synthetic data generation uses a similar technique in which the mean and covariance matrix is shared between both classes. Both these approaches have been elaborated on in the next part of the paper.

### Feature engineering and choice of optimal classifier

There have been numerous applications where engineering new features out of the existing ones can improve classification accuracy, especially when dealing with small datasets. That is why, the following four techniques of feature transformations have been used in this case:, (i) Normal Quantile Distribution on the entire dataset with the number of quantiles as 100 and the output distribution as uniform, (ii) Applying Power Transformation [37] on the numerical predictors, (iii) Applying Power Transformation on the numerical predictors and clipping the highest and lowest quantiles of data and (iv) Ranking the numerical predictors and applying Normal Distribution transformation on all the predictors.

For each of these cases, a comparative analysis of the performance of three kernel-based classifiers (Adaboost classifier, Linear Support Vector Machine with varying values of penalty parameter, and Gradient Boosting Classifier) have been tested by dividing the data into five splits and repeating the process fifteen times to decide the optimal classifier. Based on the chosen classifier, a set of rules have been prescribed to regulate the nutrient concentrations in the aquaponic solution for optimal growth of both fish and plants in a unified set-up.

## Results

The initial size of the dataset used in this study was 201 observations x 32 predictors. However, as stated before, the size of the dataset was reduced to 7 predictors before comparing classifier performance. To begin with the analysis, a pairwise correlation matrix [38] was constructed using Factor Analysis [39], and the list of predictors which were removed for having more than 90% correlation between them are as follows: Magnesium (ppm), Hardness (grains CaCO3/gallon), Hardness (ppm CaCO3), Alkalinity (ppm CaCO3), Total Dissolved Salts (ppm) and Copper (ppm).

After this, ExtraTreesClassifier [40] with 250 estimators and a depth of 5 was applied to the numerical predictors in the dataset to find out the feature importance and eliminate the predictors which contributed less than 5% importance in the analysis. The importance of each numerical feature has been stated in Table 2.

**Table 2:** Feature importance values of the predictors in the analysis given by the ExtraTreesClassifier.

Sl. No.	Name of the Predictor	Feature Importance (%)
1	Calcium (ppm.)	3.4
**2**	**Sodium (ppm.)**	**8.42**
3	Potassium (ppm.)	3.34
4	Boron (ppm.)	3.37
**5**	**Bicarbonate (ppm.)**	**8.80**
6	Sulfate (ppm.)	3.58
**7**	**Chloride (ppm.)**	**9.28**
**8**	**Nitrate-N (ppm.)**	**5.13**
9	Phosphorus (ppm.)	3.32
**10**	**pH**	**8.79**
11	Conductivity (umhos/cm)	4.78
**12**	**SAR**	**6.40**
13	Iron (ppm.)	3.68
14	Zinc (ppm.)	4.35
15	Manganese (ppm.)	4.68
16	Charge Balance	2.65
17	Temperature (K)	3.68
18	Humidity (%)	4.79
**19**	**Wind speed (mph)**	**5.77**
20	Pressure	0.73
21	Precipitation	0.96

Based on the features that have been highlighted in the above table, a decision was taken to remove the following predictors from the analysis as they yielded less than 5% importance: Calcium (ppm), Potassium (ppm), Boron (ppm), Sulfate (ppm), Phosphorus (ppm), Conductivity (umhos/cm), Iron (ppm), Zinc (ppm), Manganese (ppm), Charge Balance, Temperature (K), Humidity (%), Pressure (mm) and Precipitation (inch). After removing these 14 predictors, there were a total of 7 numerical predictors on which RFE with XGBoost classifier was used to select the top three numerical predictors. Therefore, the final set of numerical predictors used in the analysis were Sodium (ppm), Bicarbonate (ppm), and Chloride (ppm) to which four categorical predictors were appended namely the one-hot encoded PLACE_CLASS storing the county in which the observations were recorded and the other storing the month when these observations were taken.

Next, the Synthetic Minority Overestimation technique with a 3-NN classifier has been used to create a balance between both the classes in the dataset before generating synthetic data. The first case of synthetic data generated using the MC technique involves the creation of a different Mean and Covariance matrix between both the classes and has been shown in Table 3.

**Table 3:** Synthetic data generation using the MC technique where the mean and covariance matrices are not shared between the classes.

Sl. No.	Class	Month Class	Place Class	Original Number of Datapoints in the Dataset	Number Of Synthetic Datapoints generated
1	1	4	0	12	50
			1	10	40
			2	25	100
2	1	3	0	3	12
			1	0	0
			2	5	20
3	0	0	0	30	120
			1	15	60
			2	45	190
4	0	1	0	11	44
			1	8	32
			2	21	84
5	0	2	0	7	28
			1	6	24
			2	3	12
6	0	3	0	0	0
			1	0	0
			2	0	0
7	0	4	0	0	0
			1	0	0
			2	0	0

Therefore, the total number of synthetic data points generated with different mean and covariance matrices between the classes is 816. Likewise, as stated above, the second case of the MC approach takes into account a shared mean and covariance matrix between both the classes and the generation of synthetic data using that approach has been stated in Table 4.

**Table 4:** Synthetic data generation using the MC technique where the mean and covariance matrices are not shared between the classes.

Sl. No.	Month Class	Place Class	Original Number of Datapoints in the Dataset	Number Of Synthetic Datapoints generated
1	0	0	29	116
		1	15	60
		2	46	184
2	1	0	11	44
		1	8	32
		2	21	84
3	2	0	7	28
		1	6	24
		2	3	12
4	3	0	3	12
		1	0	0
		2	5	20
5	4	0	12	48
		1	10	40
		2	25	100

As observed from the above table, the number of synthetic data points generated with shared mean and covariance matrices between the classes is 804. This takes the total size of the dataset to 1620 observations x 7 predictors which have been used in the next part of the paper for analysis.

**Fig 2:**
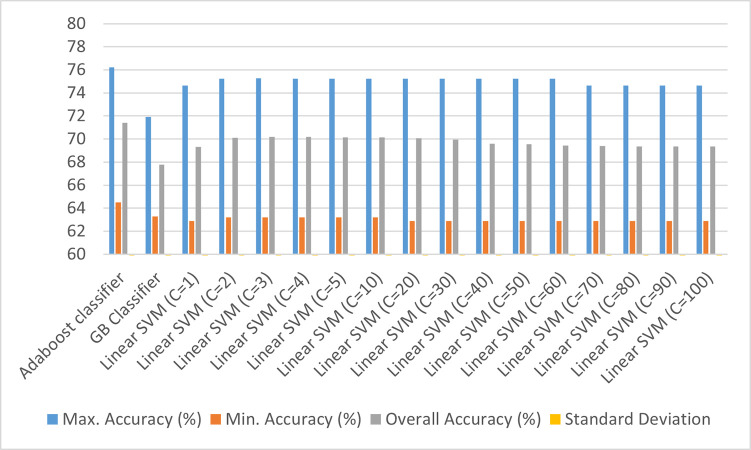
Classification results using baseline model (without any feature engineering techniques).

**Fig 3:**
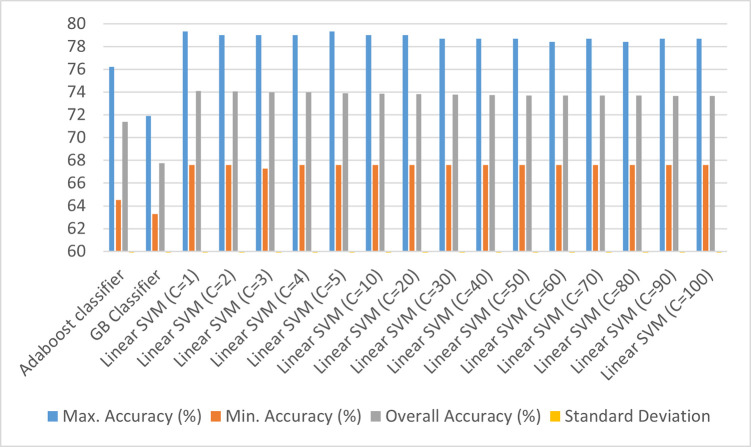
Classification results using quantile transformation on the dataset (applying normal quantile distribution on the dataset with the number of quantiles set to 100 and output distribution as uniform).

**Fig 4:**
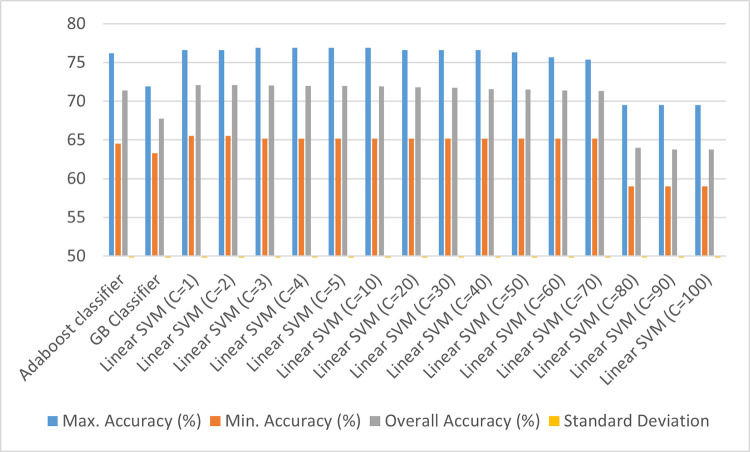
Classification results using power transformation on the numerical predictors and later appending the categorical predictors to the dataset.

**Fig 5:**
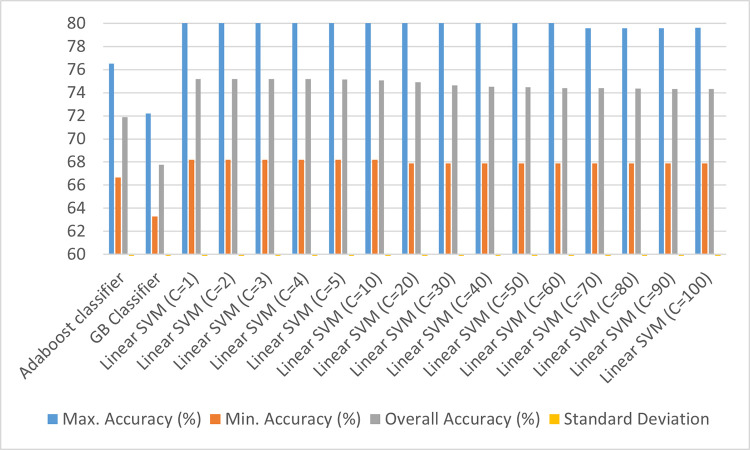
Classification results using power transformation on the numerical predictors and clipping the lowest and highest quantiles of data.

**Fig 6:**
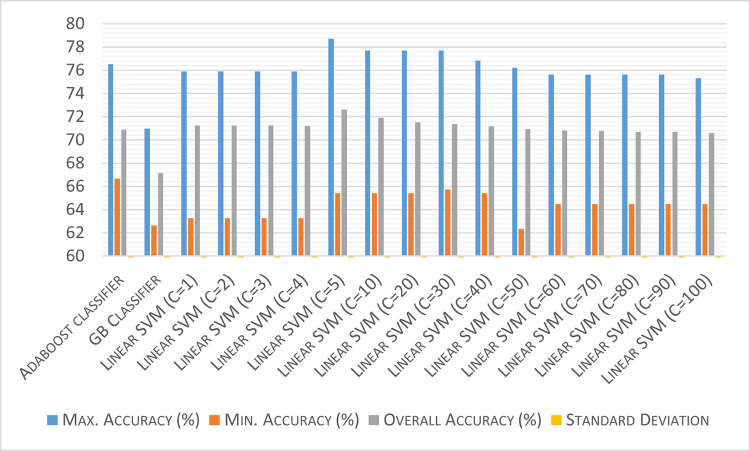
Classification results using gaussian transformation on the dataset after ranking the numerical predictors.

As discussed above, feature engineering may play an important role when inferencing with small datasets. That is why, a comparative study of the four feature engineering techniques on the deterministic classifiers (Adaboost, Linear SVM with varying values of penalty parameter (C), and Gradient Boosting classifier (GB classifier)) have been done in reference to the baseline model [Figs 2–6]. For these techniques, each classifier is trained and tested on 5 splits of data with 15 repeats, and the aggregate testing accuracy is recorded. Based on the results, the ideal Feature Engineering technique with the classifier has been chosen to suggest recommendations for the prescribed set-up. The Standard Deviation observed in case of each of the classifiers is 0.02 at maximum which is not significant from a statistical perspective.

Comparing the deterministic models at hand, it seems that Linear SVM went on to perform well on the dataset with minimum Standard Deviation between its accuracies. However, with maximum accuracies hovering around 70% for the classifiers, the predictors have been transformed using various Feature Engineering techniques which have been discussed in Figs 3–6.

From Fig 3, it can be stated that the overall accuracies for all the Adaboost classifier and Linear SVM with different values of penalty vary between 70 to 75%, except for Gradient Boosting classifier which has an aggregate accuracy of 68%. In order to improve the classification accuracy, the variance between the numerical predictors is stabilized so that the output distribution is more normally distributed which also improves the Pearson correlation among the variables. This has been addressed in Fig 4.

From Fig 4, it can be observed that the aggregate accuracies for all the three classifiers do not show any significant improvement in classifier performance, with even further degradation when the value of penalty parameter for the Linear SVM is increased beyond 70. To address this, there has been an attempt to remove the outliers in the dataset by clipping the lowest and highest quantiles of data. The classification results of the analysis have been included in Fig 5.

In the last method of data transformation, the numerical predictors i.e. Sodium (ppm), Bicarbonate (ppm), and Chloride (ppm) are assigned a rank by the algorithm, and then a Gaussian Quantile Transformation is applied to the entire dataset with the number of quantiles as 1000 and output distribution as uniform which has been stated in Fig 6 below.

Therefore, based on the above simulation results, a decision was taken to proceed with power-transforming the numerical predictors in the dataset namely, Sodium (ppm), Bicarbonate (ppm), and Chloride (ppm) so that the variance is reduced between the predictors. The lowest and the highest quantiles of the data were also clipped so that the outliers are removed from the dataset. After this, a Linear SVM classifier with a penalty parameter set to 1 was decided as the ideal classifier in this case as it yielded 75.18% aggregate accuracy on the test set with 5-fold cross-validation, with the algorithm repeated for 15 times.

## Discussion

**Fig 7:**
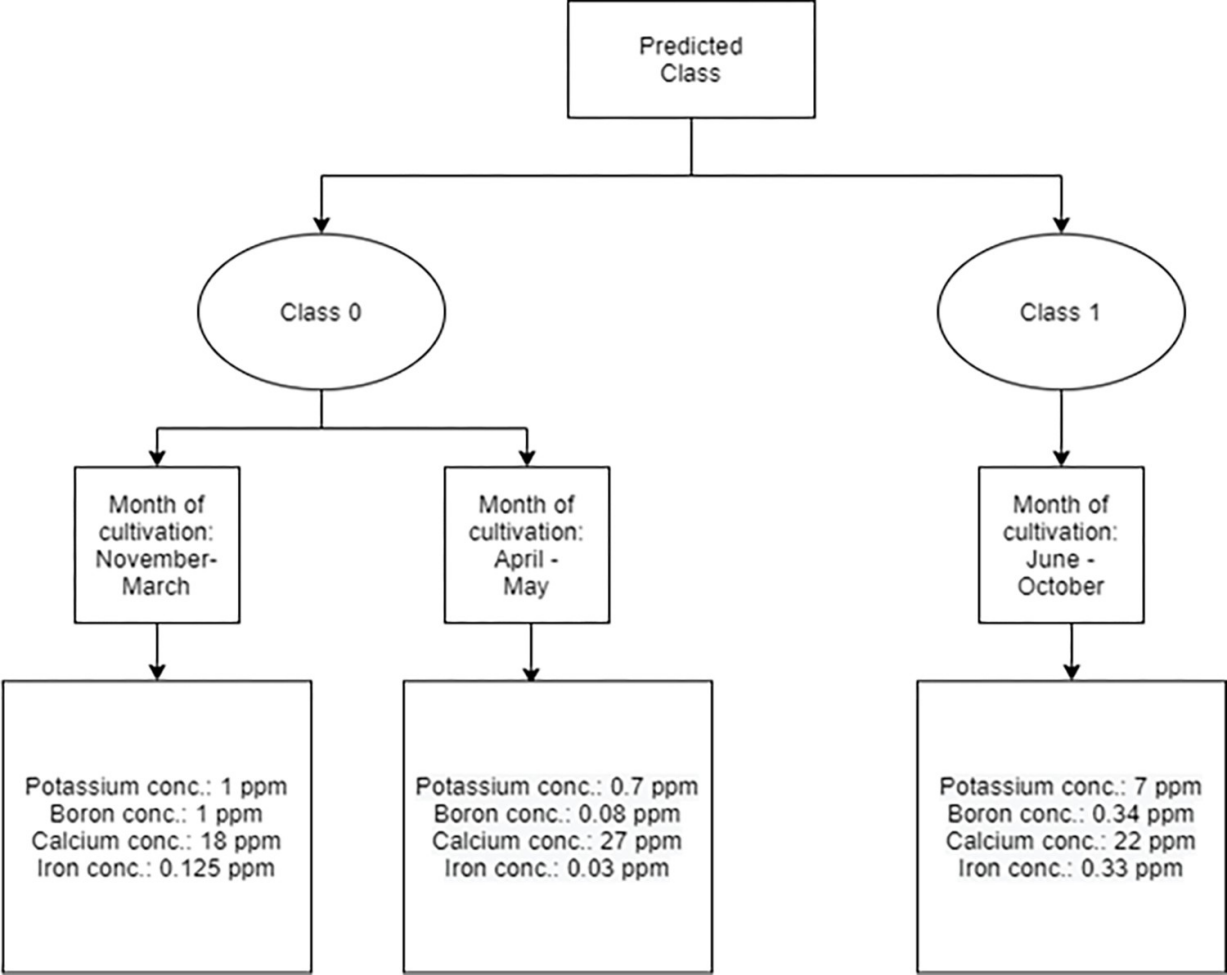
Decision tree stating the recommended rules based on the output from the Machine Learning system.

As described above, a set of rules have been recommended for each class, which are shown in the decision tree schematic in Fig 7.

The decision tree stated above recommends a system based on the output of the Machine Learning model trained on historical data that has been collected for a year. It states the appropriate concentration of nutrients that should be maintained in the aquaponic solution based on the time of the year for optimal growth of plants and fish in the integrated set-up.

Based on the above results, if the predicted class is 0 and the month of cultivation varies between November and March (peak winter months) and April to May (spring and early summer months), the potassium concentration in the aquaponic solution is maintained at about 1 ppm as it is an important nutrient for protein synthesis in plants [41]. Similarly, if the predicted class is 1 and the months of cultivation are from June to October (peak summer months and onset of fall season), the potassium concentration is increased to 7 ppm to make up for the loss of nutrients from the aquaponic solution due to evaporation. Further elaborating on this, potassium plays an important role in the nitrogen metabolism of plants and maintaining the root-shoot ratio, net photosynthetic rate, and root activity. Therefore, considering the historical data, maintaining the abovementioned concentration of potassium is vital to sustain plant growth. For the growth of fish in the system, the concentration of potassium is not a limiting factor since the amount of potassium provided through fish feed would be enough to sustain fish growth [42].

As the plants grown here complete one growth phase in 21 days, Boron concentration needs to be maintained at the levels stated above to ensure optimal root growth of plants. For class 0, the recommended level of Boron for plants grown from November to March is 1 ppm. This is because most of the green leafy vegetables are grown during these months and having a high concentration of Boron is a must for cell wall formation and stability, maintenance of structural and functional integrity of biological membranes, and movement of sugar or energy into growing parts of plants [43]. Maintaining an optimum concentration of Boron is also important for the uptake of Potassium and Phosphorus, which are two important macronutrients for plant growth. Maintaining a high concentration of Boron is reported to be important for stimulating fish growth as well [44].

The recommended concentrations of Calcium for each of the predicted classes are shown in Fig 1. If the predicted class is 0 and the months of cultivation are from April to May, then the concentration of Calcium is maintained at 27 ppm since it is the period when tomatoes begin ripening, Calcium deficiency at this stage is known to result in blossom-end rot [45]. Except for this period, the concentration of Calcium is maintained at a moderate level for the healthy growth of leafy vegetables, as shown in Fig 2. For fish growth, maintaining an optimal concentration of Calcium is important for their skeletal development throughout the course of their life cycle [46], and recommended Calcium levels are optimal depending on the months in which fish is cultivated.

If the predicted class is 1 and the time of cultivation is the peak summer months or the early part of the fall season, the Iron concentration in the aquaponic solution is maintained at a higher level due to the rapid rate of evaporation, which may happen during the season. This is important as Iron deficiency would result in a lack of chlorophyll production, resulting in poor crop yield and quality, and an increase in chances of bacterial infection [47]. For fish growth, Galbraith et al. [48] reported that the addition of ferrous compounds resulted in a sharp decline in the mortality rate of fish from hatching to maturity. Iron supplementation in the aquaponic solution could likely have improved overall fish survival in the current study, but it was not explicitly studied.

The main advantage of the decision tree developed here is to provide a recommendation system that is dependent on a few basic parameters of the aquaponic solution i.e. sodium, bicarbonate, and chloride concentrations which are given as input parameters in the Machine Learning algorithm, and based on the month in which the observations are recorded, the model outputs an appropriate concentration of Potassium, Boron, Iron and Calcium that should be maintained in the aquaponic solution. In aquaponics, the lack of technologies for the automation of nutrient application has long been a limitation for improving efficiency to support wider adoption. This work is an improvement over the existing research in this field which mostly focuses on monitoring plant and fish growth in a controlled set-up. This work is the first of its kind to propose a recommendation system for automatically controlling nutrient concentrations in aquaponics to be used on a commercial scale.

However, a system based on these recommendations is yet to be implemented on a commercial scale to prove its efficacy. The current approach takes into consideration the method of inferencing using Machine Learning models which are trained and tested on a synthetic dataset. In the future, more efforts need to be made on devising techniques for inferencing with limited original data rather than generating synthetic data. An actuation set-up can also be built based on these recommendations for real-time monitoring and regulation of these nutrient concentrations in aquaponic solutions.

## Conclusion

From the above experimental results, it can be concluded that to predict the optimal nutrients required for fish and plant growth in a single aquaponic set-up, Monte-Carlo (MC) techniques have been used for synthetic data generation, followed by power-transforming the numerical predictors and clipping the highest and lowest quantiles of data as feature engineering methods. Based on the data that was used to design the approach, Linear Support Vector Machine with penalty parameter set to 1 was chosen as the ideal classifier as it yielded more than 75% accuracy on the test data set. A set of recommendation rules have been prescribed in the discussion on how certain concentrations of nutrients in the aquaponic solution are regulated based on the predicted class and the month in which the plants are grown. In addition to this, this paper can also be used for designing approaches for other domains with sparse data.
